# Hygric Behavior of Viticulture By-Product Composites for Building Insulation

**DOI:** 10.3390/ma15030815

**Published:** 2022-01-21

**Authors:** Céline Badouard, Chadi Maalouf, Christophe Bliard, Guillaume Polidori, Fabien Bogard

**Affiliations:** 1 MATériaux et Ingénierie Mécanique, MATIM, University of Reims Champagne Ardenne, CEDEX 2, 51687 Reims, France; chadi.maalouf@univ-reims.fr (C.M.); guillaume.polidori@univ-reims.fr (G.P.); fabien.bogard@univ-reims.fr (F.B.); 2 Institut de Chimie Moléculaire de Reims, ICMR-UMR 7312 CNRS, University of Reims Champagne Ardenne, CEDEX 2, 51687 Reims, France; christophe.bliard@univ-reims.fr; 3 Pôle de Recherche Châlonnais, University of Reims Champagne Ardennes, 51000 Châlons-en-Champagne, France; 4 SFR Condorcet—FR CNRS 3417, Centre de Recherche en Environnement et Agronomie (C.R.E.A.), 2 Esplanade Roland Garros, 51100 Reims, France

**Keywords:** bio-based composite, agromaterials, hygric properties, renewable raw sources, hygrothermal performance

## Abstract

One possible approach to reducing the environmental impacts associated with the building sector is the development and use of bio-based building materials. The objective of this study is to determine the water properties of bio-based insulation materials, derived from winegrowing co-products, which promote energy efficiency. The water performance of these new bio-based materials is based on the measurement of the moisture buffer value, the sorption isotherm, and the water vapor permeability. Four by-products are analyzed: stalks, grape pomace, crushed stalks, and skins; they are combined with a potato starch binder. The performance of these composites is compared to two other bio-based composites (hemp/starch and beet pulp/starch). The stalk/starch composite can be classified as a hygroscopic and breathable material with excellent moisture retention capacity.

## 1. Introduction

The construction industry is the largest energy consumer in the European Union (about 31% of total energy consumption) and the main sector responsible for greenhouse gas emissions (about 23% of total EU carbon dioxide emissions) [1]. The construction sector has a crucial role to play in helping to achieve the target of a 55% reduction in greenhouse gas emissions by 2030 compared to the level observed in 1990.

In 2015, France committed during the Paris agreements to drastically reduce its carbon emissions to achieve carbon neutrality in the second half of this century. According to Ürge-Vorsatz et al. [2], the energy demand for heating and cooling could be reduced by almost half by 2050 compared to 2005 levels by applying today’s best energy-efficient technologies. To best respect this commitment, France has put in place a new environmental regulation for energy transition for the building sector. At the end of 2021, this new environmental regulation (RE202) will propose requirements in this direction by significantly improving the insulation performance of new buildings; by promoting low-emissive construction methods, carbon-free energies, and bio-based building materials; and, finally, by integrating a summer comfort objective to adapt buildings to global warming [3].

There is a growing interest in bio-based insulation materials because they are renewable and often easily recyclable, and, during their growth, they have sequestered large amounts of carbon from atmospheric carbon dioxide through photosynthesis. In general, the absorbed carbon dioxide is greater than the carbon dioxide incorporated into their manufacturing process. This means that their use in construction reduces the net amount of carbon dioxide incorporated into the building, which can lead, in some cases, to a “negative” carbon footprint [4,5].

The thermal conductivity of bio-based insulation materials (λ ~ 0.03–0.08 W·m^−1^·K^−1^) [6,7] is generally slightly lower than that of mineral insulation products and, in particular, rigid foams. Bio-based insulation materials have the advantage over synthetic materials of having excellent hygrothermal performance [8]. They have the ability to regulate indoor air humidity by absorbing and desorbing water vapor for extreme air relative humidities, reducing the need for ventilation (heating in winter and air conditioning in summer) and, therefore, electricity consumption. They do not contain pollutants, such as COx, NOx, and Sox; they emit very little volatile organic compounds (VOCs); and their production is not energy intensive [9].

In this work, we have chosen to study the use of widely available co-products from vine production as a potential resource for new insulation materials. The grape pomace, also called “aignes” in the Champagne-producing region (Champagne-Ardenne), corresponds to all the solid parts that remain at the end of the pressing process.

The objective of this study is to present the hydric properties of composites made from these aignes associated with potato starch. The formulations used and the thermomechanical and acoustic study of the composite thus obtained have already been reported by the authors [10].

## 2. Materials and Methods

### 2.1. Materials and Samples

**Figure 1:**
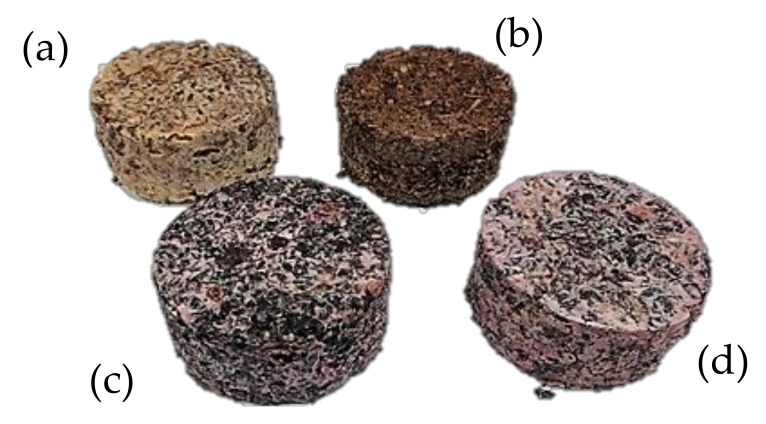
Composites (**a**) A/S, (**b**)A/CS, (**c**) A/P, and (**d**) A/GP.

The wine co-products were recovered from a local plant during harvest. Four formulations were considered for trials with four different types of aggregates: grape pomace (GP), skins (P), stalks (S), and crushed stalks (CS). The binder chosen was potato starch (A), which was supplied by Roquette (Lestrem, France). Heating the starch powder at 95 °C in water leads to irreversible gelatinization caused by swelling of the grains. The obtained gel acts as a binder of the aggregates. The starch/mass ratio in composites was set at 0.2, while the starch/water ratio was set at 0.1. The appropriate amounts of water and starch were heated at 95 °C under vigorous mechanical stirring until a translucent appearance and high viscosity were obtained. Wine by-products, grape pomace (GP), stalks (S), skins (P), and crushed stalks (CS) were mixed and kneaded with the starch gel for a few minutes in order to obtain a good homogeneity. The resulting mixtures were shaped in molds and then compacted under a pressure of 40 kPa for five minutes. The samples were frozen and then freeze dried under a pressure of 3 mbar for seven days. Figure 1 shows the composites obtained. The material density is presented in Table 1.

### 2.2. Characterizations

#### 2.2.1. Permeability 

Water vapor permeability δ_v_ (kg·m^−1^·s^−1^·Pa^−1^) is an important property of a material, as it characterizes the ability of the material to transfer moisture (diffusion, effusion, and transfer of liquid) under vapor pressure gradient. The measurement of water vapor permeability was determined by the dry cup method described in ISO 12572 [11], under isothermal conditions at 23 °C. The samples (50 cm^2^ exposed and 4 cm thick) were conditioned at 23 °C and 50% relative humidity (RH) until weight stabilization. The test was performed on three samples of each formulation. The results presented are averaged over these three samples for each formulation.

**Figure 2:**
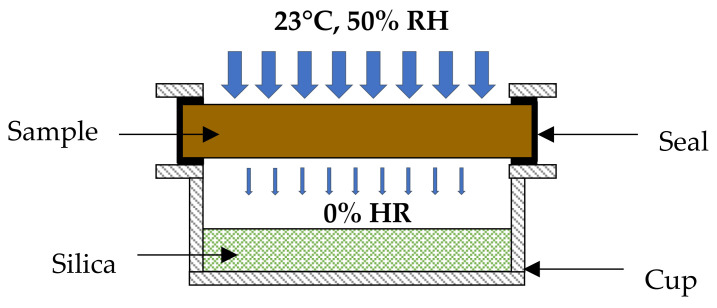
Dry cup device for measuring water vapor permeability.

To obtain the unidirectional moisture flow, the samples were sealed on the sides of the cups that contained a specific compound for moisture control (dry cup method, silica gel); Figure 2 illustrates the device used. This system kept the relative humidity of the air layer in the cup at 0%. The depth of the air layer between the sample and the silica gel was 3 cm. The sample–cup assembly was placed in a Binder MKF 720 climate chamber set at 50% RH and 23 °C. The mass of the assembly was measured daily until a variation lesser than ± 5% was obtained. 

The water vapor resistance factor, μ, and the water vapor permeability, δ_v,_ are given by Equations (1) and (2), respectively. G is the mass flow rate (kg·s^−1^), ΔP_v_ is the water vapor pressure gradient, d is the thickness of the sample (m), *A* is the exposed area (m^2^), and δ_a_ is the water vapor permeability of the air (kg·m^−1^·s^−1^·Pa^−1^). The equivalent air layer depth for water vapor diffusion S_d_ is given by Equation (3), where d is the width of the sample in m.

(1) $$\delta_{v}=\frac{G\times d}{\Delta P_{v}\times A}$$

(2) $$µ=\frac{\delta_{a}}{\delta_{v}}$$

(3) $$S_{d}=\mu \times d$$

#### 2.2.2. Sorption Isotherm

The hygroscopic curve describes the balance between the water content of the material studied and the relative humidity of the ambient air. The desiccator method described in ISO 12751 [12] was used for this study. These results allowed us to draw a sorption curve representing the variation in the water content as a function of the relative humidity of the ambient air at a constant temperature of 20 °C. The samples were dried for 24 h in an oven at 50 °C to extract the residual moisture. The test was carried out on three cylindrical samples with a diameter of 10 cm and a thickness of 2 cm for each formulation. The results presented are averaged over these three samples for each formulation. To determine the adsorption isotherm, the samples were successively exposed to increasing humidity levels of 22, 33, 55, 75, and 93%.

For each RH level, samples were weighed periodically until they reached a constant mass (i.e., the mass change between three consecutive weighings, performed at least 24 h apart, was less than 0.1% of the total mass). The water content was calculated using Equation (4):

(4) $$u=\frac{m_{i}-m_{0}}{m_{0}}=\frac{m_{w}}{m_{0}}$$

where m_0_ (g) is the dry mass of the sample, m_i_ (g) is the mass of the sample at a given relative humidity, and m_w_ (g) is the mass of the water absorbed by the sample.

The experimental results were then adjusted to three analytical models: Merakeb [13], GAB [14], and van Genuchten [15]. The equations are given, respectively, in Equations (5)–(7):

(5) $$\mathrm{Merakeb}:\;\ln\left(\frac{u}{u_{s}}\right)=a\times \ln\left(\varphi \right)\times e^{b\varphi }$$

where u is the moisture content by mass, u_s_ is the saturation moisture content by mass, φ is the relative humidity, and a and b are the Merakeb model constants.

(6) $$\mathrm{GAB}:\;W=\frac{W_{m}\times C_{G}\times K\times \varphi }{\left(1-K\times \varphi \right)\;\left(1-K\times \varphi +C_{G}\times K\times \varphi \right)}$$

where W is the moisture content by mass, W_m_ is the saturation moisture content by mass, φ is the relative humidity, and K and C_G_ are the GAB parameters.

(7) $$\mathrm{van}\;\mathrm{Genuchten}:\;u=u_{s}\times \left(1+{\left|\frac{\alpha_{T}\times 8,314\times 296\times \ln\left(\varphi \right)}{0.018\times 9.81}\right|}^{\eta T\left(-1+\frac{1}{\eta T}\right)}\right)$$

where u_s_ is the saturation moisture content by mass, φ is the relative humidity, and α_T_ and η_T_ are the van Genuchten parameters.

Experimental data were correlated with the least squares method. To estimate the variability assigned to each model, the correlation coefficient (R^2^) was calculated. Mean deviation E and mean quadratic error (RMSE) were also assessed. These criteria allowed us to assess how the model fitted the experimental results.

#### 2.2.3. Moisture Buffer Value (MBV)

MBV represents the ability of the composite to regulate the relative humidity of a medium. The Nordtest protocol [16] defines the cyclical variations in relative humidity after stabilization, between high (75%) and low (33%) values for 8 h and 16 h. The test was carried out on three cylindrical samples with a diameter of 10 cm and a thickness of 2 cm for each formulation. The results presented were averaged over these three samples for each formulation.

The edges and back sides of the samples were sealed with waterproof adhesive tape. The samples were stabilized at 23 °C and 50% RH in a climate chamber and weighed five times during the absorption phase and two times during the desorption phase. This test was performed with an air velocity equal to 0.5 m/s. The water buffer capacity MBV was calculated using Equation (8):

(8) $$\mathrm{MBV}=\frac{\Delta m}{A\times \left({\mathrm{RH}}_{\sup}-{\mathrm{RH}}_{\inf}\right)}$$

where A (m^2^) is the surface of the sample in contact with air. RH_sup_ and RH_inf_ represent, respectively, the high relative humidity (75% RH) and the low relative humidity (33% RH), and Δm represents the mass variation during the adsorption phase (g).

## 3. Results and Discussion

### 3.1. Permeability

**Figure 3:**
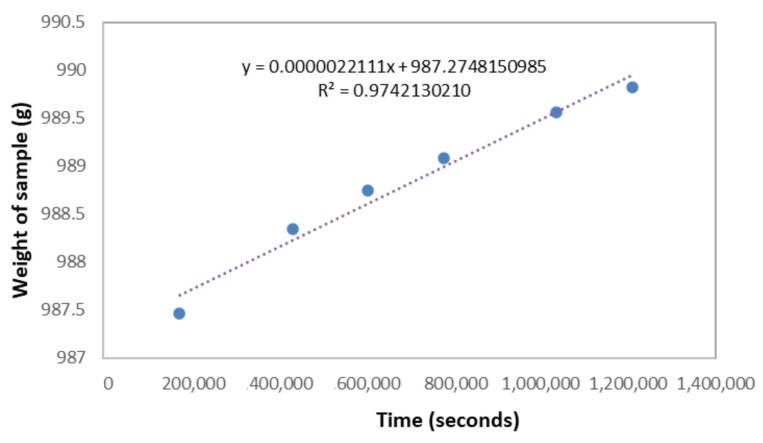
Example of diffusion flow stabilization in a dry cup test of an A/P sample to determine diffusion properties and the linear regression between the mass of the assembly and time with the obtained equation (dotted line).

The water vapor permeability results are presented in Table 2. The stabilization of the diffusion flow during the dry cup test was almost linear for all samples of each composition (see Figure 3). The mass flow rate G (kg·s^−1^) from Equation 1 was obtained by taking the slope of the regression line between the mass of the sample–cup assembly and the time shown in Figure 3.

The resistance factor to water vapor diffusion μ was between 11.2 and 13.4. The water vapor permeability, δ_v_, was between 1.40 × 10^−11^ kg·m^−1^·s^−1^·Pa^−1^ for the stalk composite (A/S) and 1.79 × 10^−11^ kg·m^−1^·s^−1^·Pa^−1^ for the grape pomace composite (A/GP). The values obtained in this study are intermediate between the hemp/starch (H/S) composite [17] and the beet pulp/starch composites (BP/S) [18]. In comparison to the composite with the same polymer matrix (starch), the water vapor diffusion resistance factor μ is 25.7 and the water vapor permeability is 0.78 × 10^−11^ kg·m^−1^·s^−1^·Pa^−1^ for BP/S, while for H/S, δ_v_ is equal to 4.74 × 10^−11^ kg·m^−1^·s^−1^·Pa^−1^, and µ is 4.22. The nature of the aggregates seems to have an impact on the permeability and vapor resistance of the composites. 

The water vapor permeability tended to increase with the increase in material density. The lower the Sd (water vapor diffusion) value, the higher the water vapor migration in the material. Materials with an S_d_ of less than 4 cm have a good water vapor permeability, and they are open to water vapor diffusion. 

### 3.2. Sorption Isotherm 

**Figure 4:**
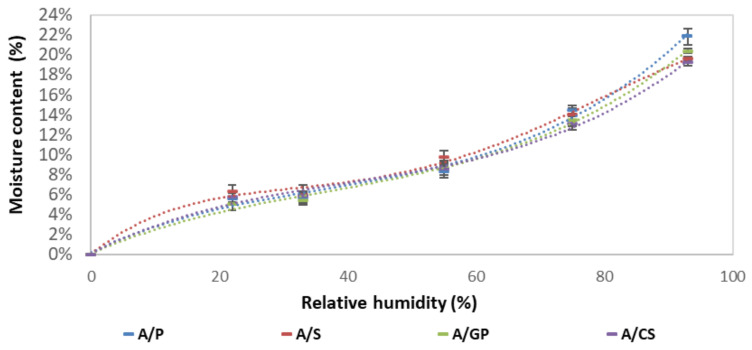
Sorption isotherms of the four composites.

Figure 4 shows the isothermal sorption curves of the four formulations studied. These curves describe the balance between the relative humidity and the moisture content of the samples at a fixed temperature of 20 °C. All isotherms have a “sigmoidal” look typical of cellulosic (and starch) materials [19,20]. This isothermal appearance is observed when the interactions between the penetrant (water) and polymer (starch) are strong. The water molecules enter the network of the material and display three distinct sorption mechanisms depending on the relative humidity level. 

At a low RH (<25%), monomolecular absorption, water vapor molecules are adsorbed and gradually cover a layer on the surface of the pores where they are held by Van der Waals forces. When the relative humidity of the air increases, medium RH (between 25 and 40%), a second layer is adsorbed. Moisture transfer occurs simultaneously through the transfer of liquid water and vapor. Finally, at a high RH (>40%), the multilayers join to form a liquid bridge separated from the gas phase by a meniscus. The water is retained on the surface of the pores by capillary forces and, thus, leads to the filling of the finest pores initially and then the largest pores by capillary condensation [21].

**Figure 5:**
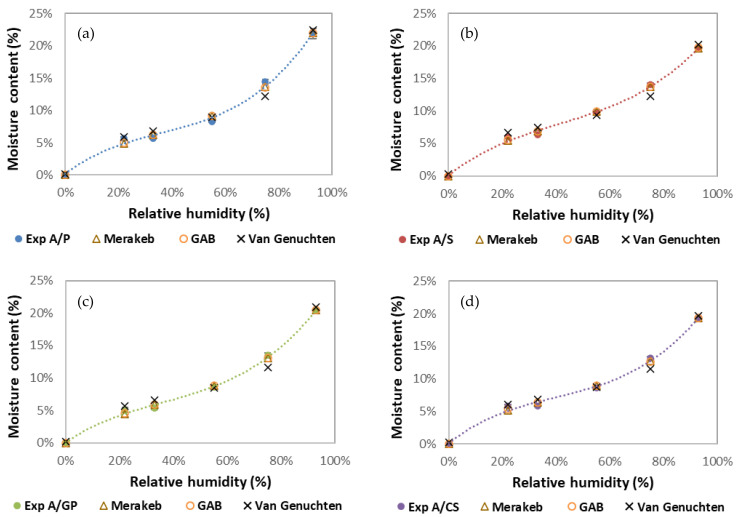
Sorption isotherms of the four composites and comparison of experimental data with Merakeb, GAB, and van Genuchten models: (**a**) A/P, (**b**) A/S, (**c**) A/GP, and (**d**) A/CS.

Figure 5a–d show the experimental values adjusted according to the GAB, Merakeb, and van Genuchten models. Of these models, the van Genuchten model appears to be the least close to the experimental values. Table 3 shows the model parameters used to adjust the sorption isotherm to 20 °C for the different formulations. For the GAB and Merakeb models, the E values are less than 10, and the correlation coefficients for all the models used are close to 1. Thus, the GAB and Merakeb models are considered appropriate [22].

As starch is very hydrophilic, it behaves similar to a sponge and is capable of absorbing water from the ambient atmosphere [23], making starch-based materials highly hygroscopic. For example, beet pulp/starch materials [18] adsorb up to 20% of water and hemp/starch materials [24] adsorb up to 17% of water at 93% relative humidity. Under very high relative humidity (93% RH) and at room temperature (20 °C), the degradation of the composite and the development of fungi on its surface are observed.

### 3.3. Moisture Buffer Value (MBV)

**Figure 6:**
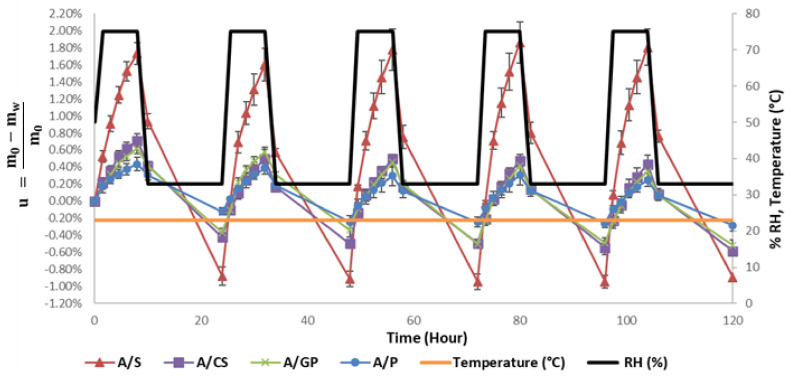
Moisture absorption cycle and desorption of the four composites during the cyclic variation of the relative moisture.

This dynamic characterization of the water buffering capacities of the materials pretends to simulate in a realistic way the daily climatic atmosphere change in a house and, thus, translates the capacities of the regulation of the relative humidity in the material. Figure 6 shows the moisture content, u, of the composites during changes in relative humidity between 33% and 75% at 23 °C, where m_w_ is the mass of the water absorbed or released and m_0_ is the initial mass of the sample. Figure 6 shows the ability of the composites to absorb moisture at 75% RH and release moisture at 33% RH. For the last three cycles, the mass variation of the samples seems to stabilize, and the final MBV is calculated (Table 4). 

The performance classification of the water buffer values according to the Nordtest project by Rode [25] is divided into five classes: negligible between 0 and 0.25 g·m^−2^·% RH^−1^, limited between 0.25 and 0.5 g·m^−2^·% RH^−1^, moderate between 0.5 and 1 g·m^−2^·% RH^−1^, good between 1 and 2 g·m^−2^·% RH^−1^, and excellent when the MBV value is higher than 2 g·m^−2^·% RH^−1^. The results presented in Table 4 clearly show that stalk, crushed stalk, and grape pomace composites can be classified as excellent environmental relative humidity regulators according to this classification. The stalk composite shows an MBV value of 6.3 g·m^−2^·% RH^−1^, which is well above the minimum of 2 g·m^−2^·% RH^−1^ for this category. However, the skin composite is only classified as a good moisture regulator. In comparison with bio-based materials with a starch binder, the samples of beet/starch pulp [18] have MBV values between 2.6 and 2.8 g·m^−2^·% RH^−1^. Hemp/starch composites [17] have similar MBV values of 2.5–2.7 g·m^−2^·% RH^−1^.

The four composites are of a different composition and structure, which can explain the great difference between them, and the proportion of starch is identical. In addition, Brouard [26] has shown that the density of aggregates has an influence on MBV. Low-density aggregates have a better MBV. In this study, the stalk composite has a density of 227 kg·m^−3^, while the skin composite is at 433 kg·m^−3^. For the A/P formulation, the material tends to crumble on the sides and can generate differences in density, which somewhat explain the high standard deviations. 

## 4. Conclusions

The characterization tests carried out on the “aignes” (grape pomace)/starch gel composites allowed us to highlight the properties of water on the various composites studied. The water vapor diffusion resistance factor was found to be between 11.2 and 14.3, indicating good permeability for the stalk composite. The moisture storage isotherms revealed the hygroscopic nature of all composites. The moisture buffer capacity (MBV) values ranged from 1.9 to 6.3 g·m^−2^·% RH^−1^. The stalk, grape pomace, and crushed stalk composites were above 2 g·m^−2^·% RH^−1^, which makes them excellent water regulators according to the NordTest project.

In general, these composites present interesting hydric properties. In comparison to other bio-based materials, our new composites have similar properties. They have the capacity to moderate the humidity of the interior air by absorbing and desorbing water vapor, which reduces the ventilation rate and, therefore, the need for heating in winter and air conditioning in summer. However, moisture is known to promote the growth of micro-organisms, discomfort, and also the degradation of the thermal stability and performance of materials. Current studies are being conducted on the durability of the composites (fungal evolution, water immersion, and fire test). Furthermore, surface treatments based on linseed oil or grape seed oil are under study in order to create microbial degradation resistance and make these materials usable in practice in the building sector.

## Figures and Tables

**Table 1:** Bulk density of composites with 20% starch.

Composite	Abbreviations	ρ_bulk_
		(kg·m^−3^)
Starch/grape skin	A/P	433 ± 78
Starch/grape pomace	A/GP	308 ± 32
Starch/stalk	A/S	227 ± 47
Starch/crushed stalk	A/CS	345 ± 35

**Table 2:** Permeability and vapor resistance of the four composites (mean ± SD).

Composite	δv × 10^−11^	µ	Sd
	(kg·m^−1^·s^−1^·Pa^−1^)		(m)
A/P	1.57 ± 0.16	12.83 ± 1.38	0.57 ± 0.06
A/GP	1.79 ± 0.12	11.22 ± 0.70	0.51 ± 0.02
A/S	1.40 ± 0.15	14.37 ± 1.59	0.66 ± 0.06
A/CS	1.65 ± 0.21	12.30 ± 1.69	0.58 ± 0.09

**Table 3:** Parameter values for sorption isotherm models.

Models	Parameters	A/P	A/S	A/GP	A/CS
Merakeb	us	0.2771	0.2322	0.2519	0.2375
	a	0.8408	0.7298	0.8516	0.7297
	b	1.4326	1.2214	1.3056	1.4581
	E (%)	7.7686	4.1947	5.3678	5.6578
	R^2^	0.9961	0.9985	0.9983	0.9979
	RMSE	0.0062	0.0034	0.0037	0.0042
GAB	Wm	0.0557	0.0664	0.0581	0.0539
	Cg	13.0356	12.3401	9.0035	22.285
	K	0.8099	0.7267	0.7825	0.7809
	E (%)	7.5877	4.1004	5.4752	5.1060
	R^2^	0.9960	0.9985	0.9983	0.9979
	RMSE	0.0063	0.0034	0.0038	0.0040
van Genuchten	Us	0.0015	0.0027	0.0016	0.0021
	αT	0.0000	0.0000	0.0000	0.0000
	ηT	1.4422	1.3722	1.4330	1.3947
	E (%)	9.4271	9.3195	9.7917	7.1207
	R^2^	0.9887	0.9883	0.9893	0.9913
	RMSE	0.0106	0.0097	0.0097	0.0081

**Table 4:** Value of the average MBV over the last three cycles and classification according to the Nordtest project (mean ± SD).

Composite	MBV	Classification
	(g·m^−2^·% RH^−1^)	
A/P	1.92 ± 0.45	Good
A/GP	2.57 ± 0.22	Excellent
A/S	6.31 ± 0.60	Excellent
A/CS	3.50 ± 0.20	Excellent

## Data Availability

The data presented in this study are available on request from the corresponding author.

## Nomenclature

Roman	
a	Merakeb model constant
A	Exposed area (m^2^)
A/CS	Starch/crushed stalk composite
A/P	Starch/grape skin composite
A/GP	Starch/grape pomace composite
A/S	Starch/stalk composite
b	Merakeb model constant
BP/S	Beet pulp/starch composite
C_G_	GAB parameters
d	Thickness (m)
E	Mean deviation (%)
G	Mass flow rate (kg·s^−1^)
RH	Relative humidity (%)
H/S	Hemp/starch composite
HR_inf_	Low relative humidity (%)
HR_sup_	High relative humidity (%)
K	GAB parameters
m_0_	Dry mass of the sample (g)
m_i_	Mass of the sample at a given relative humidity (g)
m_w_	Mass of the water absorbed by the sample (g)
MBV	Moisture buffer value (g·m^−2^·% RH^−1^)
R^2^	Correlation coefficient
RMSE	Mean quadratic error
S_d_	Equivalent air layer depth for water vapor diffusion (m)
u	Moisture content (%)
u_s_	Saturation moisture content (%)
W	Moisture content (kg)
W_m_	Saturation moisture content (kg)
Δm	Mass variation during the adsorption phase (g)
ΔP_v_	Water vapor pressure gradient (Pa)
Greek	
α_T_	van Genuchten parameters
δ_a_	Water vapor permeability of the air (kg·m^−1^·s^−1^·Pa^−1^)
δ_v_	Water vapor permeability (kg·m^−1^·s^−1^·Pa^−1^)
η_T_	van Genuchten parameters
μ	Water vapor resistance factor
φ	Relative humidity (%)
